# The induction of the p53 tumor suppressor protein bridges the apoptotic and autophagic signaling pathways to regulate cell death in prostate cancer cells

**DOI:** 10.18632/oncotarget.2528

**Published:** 2014-09-26

**Authors:** Lymor Ringer, Paul Sirajuddin, Lucas Tricoli, Sarah Waye, Muhammad Umer Choudhry, Erika Parasido, Angiela Sivakumar, Mary Heckler, Aisha Naeem, Iman Abdelgawad, Xuefeng Liu, Adam S. Feldman, Richard J. Lee, Chin-Lee Wu, Venkata Yenugonda, Bhaskar Kallakury, Anatoly Dritschilo, John Lynch, Richard Schlegel, Olga Rodriguez, Richard G. Pestell, Maria Laura Avantaggiati, Chris Albanese

**Affiliations:** ^1^ Department of Oncology and Lombardi Comprehensive Cancer Center, Georgetown University Medical Center, Washington, DC, USA; ^2^ Department of Pathology, Georgetown University Medical Center, Washington, DC, USA; ^3^ Massachusetts General Hospital, Boston, USA; ^4^ Georgetown University Hospital, Washington, DC, USA; ^5^ Kimmel Cancer Center, Thomas Jefferson University, Philadelphia, PA, USA; ^6^ National Cancer Institute of Egypt, Cairo, Egypt

**Keywords:** p53, apoptosis, autophagy, primary cells, prostate

## Abstract

The p53 tumor suppressor protein plays a crucial role in influencing cell fate decisions in response to cellular stress. As p53 elicits cell cycle arrest, senescence or apoptosis, the integrity of the p53 pathway is considered a key determinant of anti-tumor responses. p53 can also promote autophagy, however the role of p53-dependent autophagy in chemosensitivity is poorly understood. VMY-1-103 (VMY), a dansylated analog of purvalanol B, displays rapid and potent anti-tumor activities, however the pathways by which VMY works are not fully defined. Using established prostate cancer cell lines and novel conditionally reprogrammed cells (CRCs) derived from prostate cancer patients; we have defined the mechanisms of VMY-induced prostate cancer cell death. Herein, we show that the cytotoxic effects of VMY required a p53-dependent induction of autophagy, and that inhibition of autophagy abrogated VMY-induced cell death. Cancer cell lines harboring *p53* missense mutations evaded VMY toxicity and treatment with a small molecule compound that restores p53 activity re-established VMY-induced cell death. The elucidation of the molecular mechanisms governing VMY-dependent cell death in cell lines, and importantly in CRCs, provides the rationale for clinical studies of VMY, alone or in combination with p53 reactivating compounds, in human prostate cancer.

## INTRODUCTION

The efficacy of anti-tumor agents relies on their ability to trigger cellular programs of apoptosis, senescence and mitotic catastrophe that lead to cell death. Impairment of apoptosis confers resistance to tumor therapy and may contribute to disease progression and recurrence. Several studies suggest, however, that apoptosis *per se* is the not only, or even the predominant, mechanism of cell death during chemotherapy [1-4]. Among the alternative mechanisms, autophagy, either concomitantly with- or independently- of apoptosis, is emerging as an important pro-cell death, anti-tumor pathway.

Autophagy is a degradative process by which damaged cellular organelles and abnormally folded proteins are cleared via the lysosome [5-7]. Autophagy may participate in either tumor suppressive or collaborative oncogenic signaling [8, 9]. In normal tissues and in many tumor cells, autophagy enables adaptation during nutritional stress via the degradation of macromolecules and intracellular organelles, thereby promoting cancer cell proliferation. In contrast, the impairment of autophagy can promote malignant transformation, as the mono-allelic deletion of Beclin-1 or loss of heterozygosity of several autophagic genes occurs in human tumors [5, 10, 11]. Multiple myeloma cells succumb to excessive autophagic activation triggered by inhibition of caspase 10 [12] and, we have shown that autophagy induced by glucose restriction [13] or by inhibitors of the mitochondrial transporter SLC25A1/CIC [14] can be directly responsible for cell death.

The activity of the *p53* tumor suppressor gene is induced by a broad array of cell stressors including DNA-damaging chemotherapeutic drugs and can be an excellent target for therapeutic intervention [15]. While the role of p53 in regulating apoptosis is well documented, various lines of evidence suggest that p53 and autophagy are also closely connected, although in a complex and at times conflicting manner. Pharmacological inhibition or ablation of p53 can enhance autophagy during nutrient stress and hypoxia [8], contributing to cell survival [16]. However, autophagy stabilizes p53 [17], resulting in a feed-forward activation of p53-dependent autophagy and cell death following DNA damage. p53 can also induce autophagy via inhibiting mTOR (reviewed in [18, 19]). Understanding the role of p53 to either induce or inhibit autophagy is important in determining therapeutic outcomes and based on these and other studies, we and others have proposed that autophagy contributes to the ability of p53 to eliminate cells that have been exposed to genotoxic stressors, preserving cellular and genomic integrity [9, 20, 21].

One obstacle to the development of new prostate cancer therapeutics has been the inability to establish sustained cultures of primary normal prostate and prostate cancer cells derived from patients. We have developed a novel culture methodology, termed “conditional reprogrammed cells” (termed CRCs), that provides an epithelial cell culture environment that facilitates the bypassing of replicative senescence, with the epithelial cells becoming reversibly immortalized without detectable cell crisis [22-25]. The ability to rapidly generate primary human cell cultures provides a unique opportunity to define the genetic and molecular basis of prostate cancer and to establish a framework for the personalization of therapy. This unique approach has been integrated into the present study.

The *in vitro* [26-28] and *in vivo* [29] anti-tumor activities of a novel CDK inhibitor, VMY-1-103 (VMY), were previously described, and VMY induces p53 activity and apoptosis in the wild type p53 prostate cancer cell line, LNCaP [26]. In the present study, we sought to define the molecular and genetic mechanisms of VMY-induced cell death. Herein we show that both prostate cancer (PCa) cell lines and primary prostate cancer CRCs with wild-type p53, were highly sensitive to VMY-induced cell death and occurred via the activation of macro-autophagy. p53 null or p53 mutant cell lines were insensitive to VMY-induced cytotoxicity. Furthermore, although p53 mutant expressing cells were resistant to VMY cytotoxicity, co-treatment of these cell lines with the p53-reactivating compound PRIMA-1, which restores wild-type p53 activity, re-sensitized these otherwise resistant cells to VMY-induced cell death. Mutation of the *p53* gene occurs relatively infrequently (20%) in early stage prostate cancers but increases significantly in late stage and metastatic PCa [30]. Given that small molecules that reactivate mutant p53 are currently in clinical trials, we propose that VMY in combination with such reactivating molecules may provide a potentially effective cancer therapeutic in both the early and late stages of PCa.

## RESULTS

### VMY induces cell death in p53 wild-type cancer cell lines

**Figure 1:**
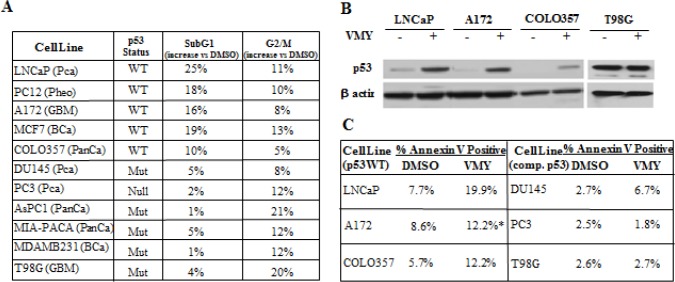
VMY activity in multiple cancer cell lines (A) The p53 status as well as the percent increases in the SubG1 and G2/M subpopulations of cells treated with 30 uM VMY for 18hrs versus DMSO are shown. Changes in cell cycle profile were assessed by flow cytometry. LnCaP and MCF7 cells and DU145 and MDAMB231cells were used as positive and negative controls, respectively, for VMY induction of cell death as previously reported [26, 28]. (B) Western blot establishing the induction of p53 protein levels by exposure to 30 uM VMY for 18hrs in the p53 wild-type human cell lines, A172 and COLO357 but not in the p53 mutant cell line T98G. LnCaP cells were used as a positive control for p53 induction as previously shown [26]. (C) Annexin V positivity as measured by flow cytometry on cells with wild-type or compromised p53 (comp. p53) followed by exposure to DMSO or 30 uM VMY for 18hrs (* 40 hours for A172). PCa, prostate cancer, Pheo, pheochromocytoma, GBM, glioblastoma multiforme, BCa, breast cancer, PanCa, pancreatic cancer.

To further clarify whether wild-type p53 is required for VMY induced cell death, we interrogated cell lines derived from a distinct tissue types and differing in p53 status. VMY-induced a G_2_/M arrest as assessed by flow cytometry, regardless of p53 status (Fig. 1A), indicating that this compound affects the cell cycle in a p53-independent manner. Many cells lacking functional p53 retain the capability to arrest in G2/M [31]. In contrast, the percentage of cells detected in the subG1 phase of the cell cycle, indicative of apoptosis, was higher in all of the cell lines harboring wild-type p53 versus cells with a p53-null or -mutant genotype. LNCaP and MCF7 and DU145 and PC3 were used as positive and negative controls, respectively [26, 32]. Consistent with our previous data [26], VMY induced wild-type, but not mutant, p53 protein levels across multiple cell lines and tissue types (Fig. 1B), which correlated with VMY-induced subG1 content (Fig. 1A).

Using flow cytometry, early apoptosis was measured by annexin V immunostaining. Propidium iodide (PI) positive, necrotic cells were excluded from these analyses. The basal levels of annexin V were uniformly higher in the p53 wild-type cells versus p53-null or p53-mutant cell lines. Treatment with VMY increased annexin positivity in p53 wild-type cells (Fig. 1C). Thus, wild-type p53 appears to be an important transducer of VMY's effects on apoptosis.

### Wild-type p53 is necessary for VMY-induced cell death

**Figure 2:**
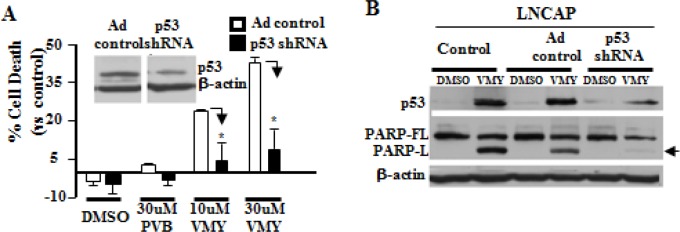
Knockdown of p53 blocks VMY-induced cell death LNCaP cells were infected with control or p53-shRNA adenovirus and incubated for 72 hrs followed by treatment with either DMSO or 30 uM VMY for 18hrs. (A) An approximate 50% reduction in p53 protein levels was observed (see inset, images are from the same autoradiogram and at the same exposure time). Cell death was significantly reduced in the p53shRNA treated cells as measured by trypan blue dye exclusion (*, *p* <0.01, N=3). (B) The effect of p53shRNA on the apoptotic machinery was confirmed by western blotting for p53 induction and for both full-length PARP (PARP-FL) and the long form of cleaved PARP (PARP-L, arrow).

To determine the role of p53 in VMY-induced cell death, an isogenic cellular system was established by using an adenoviral vector expressing p53shRNA to knock down p53 protein levels in LNCaP cells. As expected the p53-shRNA led to an approximate 50% reduction in p53 protein levels relative to control vector cells (Fig. 2A). Additionally, p53 knockdown significantly inhibited (*p*<0.01) the cell death-promoting activity of VMY as measured by flow cytometry, and nearly completely abrogated PARP cleavage induced by this compound (Fig. 2B).

**Figure 3:**
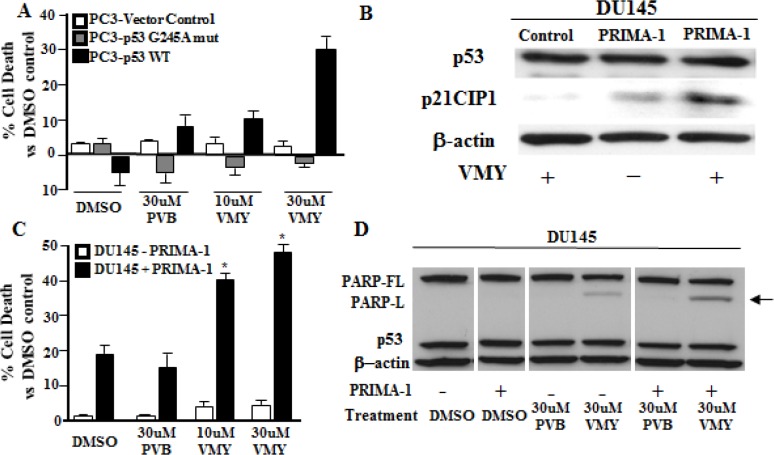
Expression of wild-type p53 but not mutant p53 restores VMY sensitivity (A) p53-null PC3 prostate cancer cells were transiently transfected with wild-type p53, p53-G245A or empty vector control. Cell viability was measured by trypan blue dye exclusion 18 hrs after treatment with VMY or purvalanol B (PVB) at the concentrations shown. (B) Western blots were performed for both p53 and for p21^CIP1/WAF1^ (p21) on extracts from PRIMA-1 and vehicle-control treated DU145 cells with or without 18 hrs exposure to 30uM VMY. (C) DU145 cells were treated with 75 uM PRIMA-1 and VMY or PVB for 18 hrs. Cell viability was measured by trypan blue dye exclusion (*, p<0.05, vs PRIMA-1 plus DMSO). (D) Western blots for p53 and the full-length PARP protein (PARP-FL) and the long form of activated, cleaved PARP (PARP-L). β-actin was used as a loading control. Western blotting was performed on extracts derived from the cells used in the experiments shown in panel C.

We next assessed the effects of expressing wild-type p53 or p53 mutants in an isogenic cellular system, thus overcoming possible differences in the genetic background. The most common p53 mutations are missense mutations occurring in the DNA binding domain and include several “hot spot” codons, including R175, G245, R248, and R273. We performed transient transfection experiments in the p53-null PC3 cells using expression vectors for WT p53 or the previously characterized gain of function p53 mutation, p53-G245A, [13, 33] (Fig. 3A). The expression of wild-type p53 decreased slightly the percent cell death in the presence of DMSO while mutant p53 expression had no effect. Consistent with our previous data (reference 26 and Fig. 2), we found that p53 negative PC3 cells are insensitive to VMY-induced cell death. Most importantly, the expression of wild-type p53 but not the p53-G245A mutant restored VMY sensitivity in these cells, leading to a progressive and dose dependent increase in the subG1 population. These results firmly establish wild-type p53 as a key effector of VMY-induced cytotoxicity

### Compounds that rescue wild-type activity of mutant p53 proteins restore sensitivity to VMY

Several small molecules including PRIMA-1 have been developed that are capable of restoring the wild-type conformation and function of mutant p53 proteins. PRIMA-1 acts via the formation of adducts with thiols contained within p53 that can rescue the biological activity of mutant forms of p53 [34]. We therefore determined whether PRIMA-1 could restore sensitivity to VMY in mutant p53 PCa cells. Treatment of the mutant p53^P223L/V274F^ DU145 human prostate cancer cell line with either VMY or PRIMA-1 alone showed only modest expression of the known down-stream p53 target gene, *p21^CIP1/WAF1^*, while co-treatment with both PRIMA-1 and VMY led to the induction of p21^CIP1/WAF1^, without affecting the total p53 expression levels, thus demonstrating restoration of p53 activity by PRIMA-1 (Fig. 3B). Importantly, co-treatment with PRIMA-1 and VMY significantly increased cell death (Fig. 3C) and induced PARP cleavage (Fig. 3D) versus either PRIMA-1 or VMY alone. All extracts were run on the same gel and the image is from one representative autoradiogram. PRIMA-1 treatment alone did not significantly affect sensitivity to VMY in either LNCaP (p53wt) or PC3 (p53-null) cells (Supplemental Fig. S1), thus demonstrating the specificity of this compound in restoring VMY-mediated cell death to mutant p53 prostate cancer cells.

### VMY induces autophagy in LNCaP cells

**Fig 4:**
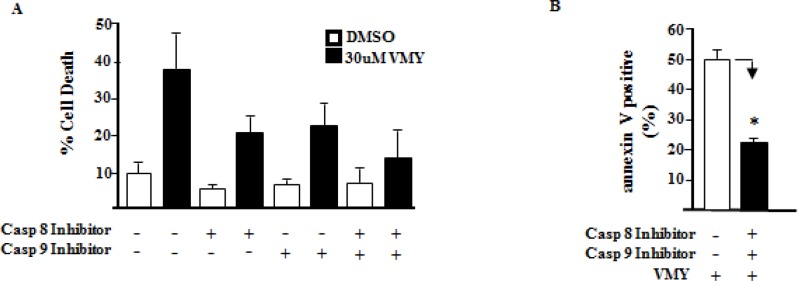
Effects of caspase inhibition on VMY-induced cell death Single and dual inhibition of caspases 8 and 9 was performed. LnCAP cells were pretreated with 20 uM of the caspase 8 inhibitor Z-IETD-FMK (Casp 8 Inhibitor), the caspase 9 inhibitor Z-LEHD-FMK (Casp 9 Inhibitor) or both, followed by treatment with 30 uM VMY for 18 hrs. (A) Cell viability as assessed by trypan blue dye exclusion. (B) The proportion of cells undergoing apoptotic cell death as assessed using annexin V staining and measured by flow cytometry. *, p< 0.05

**Figure 5:**
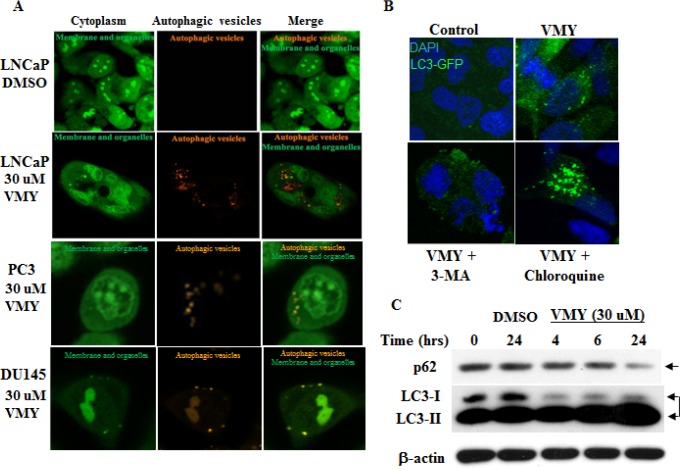
VMY induces autophagic activation in LNCaP cells (A) Photomicrographs of LNCaP, PC3 and DU145 cells stained with acridine orange (AO) and treated for 18 hrs with DMSO (top row, LNCaP) or 30 uM VMY (bottom rows). The cells were imaged on a Zeiss LSM510 Meta microscope using a 488 nm dichroic mirror and a 525/50 bandpass filter to visualize cytoplasmic- and membrane- associated AO (green, left panels) and a 700 nm short pass filter using a metadetector of 651/42 emission filter to visualize protonated AO in acidic vesicles (orange, middle panels) and as merged images (green and orange, right panels). (B) LNCaP cells were transiently transfected with LC3-GFP and treated with DMSO (top left) or VMY (top right) in the presence or absence of 5 uM 3-MA (an inhibitor of early autophagy, lower left) or 50 uM chloroquine (an inhibitor of acidification of lysosomes and autophagosomes, lower right). (C) Authophagy time-course experiments performed on LNCaP cells treated with 30 uM VMY for 4, 6 and 24 hrs. Western blotting was performed to determine the levels of the autophagic cargo receptor, p62 (arrow) and for the relative abundance of LC3I and LC3II (double arrow). β-actin was used as a loading control.

As inhibition of apoptosis with caspase 8 or 9 inhibitors failed to completely rescue VMY-induced cell death in LNCaP cells, despite a significant reduction in annexin V positivity (Fig. 4), we explored the role of autophagy in VMY-induced cell death. Autophagy was assessed by immuno-fluorescence and immuno-blot assays. The fluorescent and lysosomotropic compound Acridine Orange (AO) becomes protonated in the acidic environment of lysosomes and autophagosomes when autophagy is activated, resulting in its aggregation and accumulation. Protonated AO is identifiable via fluorescence microscopy as discrete aggregates that emit fluorescence at 640 nm, versus fluorescence at 525 nm in its native state. LNCaP cells were exposed to AO either in the presence or absence of VMY. VMY treatment resulted in the appearance of discrete acidic vesicles. We found that VMY-treated LNCaP cells exhibited a stark increase in discrete acidic vesicles with greater than 75% of the cells being AO positive, with an average of 10 AO puncta dispersed throughout the cytoplasm (Fig. 5A), thus demonstrating that VMY induces autophagosome formation. In contrast, in the few DU145 and PC3 cells that were AO positive (approximately 1%), on average only one AO puncta per cell was observed in DU145 cells and an average of two AO puncta were seen in PC3 cells (Fig. 5A)

We next studied the pattern of subcellular localization of LC3-I (microtubule-associated protein 1 light chain 3), which becomes lipidated by the class III phosphoinositide 3-kinase Vps34 when autophagy is activated and re-localizes from the microtubules to autophagosomal membranes (reviewed in Kang, et al. [35]). LNCaP, DU145 and PC3 cells were transiently transfected with an LC3-GFP expression vector [36] and subjected to fluorescence microscopy. VMY treatment induced LC3-GFP re-localization and concentration into prototypical autophagic puncta (Fig 5B, top right panel) in 11% of the LNCaP cells (with approximately 5 puncta per LC3 GFP positive cell) versus approximately 1% of VMY treated DU145 or PC3 cells with approximately 1 puncta per LC3 GFP positive cell (Supplemental Fig. S2). Treatment with VMY and 3-methyl adenine (3-MA), a class III phosphoinositide 3-kinase inhibitor, resulted in a loss of LC3-GFP puncta in LNCaP cells (Fig. 5B lower left panel, average 0.8 puncta per cell) while chloroquine, which inhibits the fusion of autophagosomes with the lysosomes, lead to an increase in the number of positive cells (20%) and an increase in the number of puncta per cell (average of more than 8 puncta per cell) (Figure 5B, lower right panel) while treatment of PC3 and DU145 cells these compounds had little effect (Supplemental Fig. S3).

Besides influencing LC3I subcellular localization, lipidation also alters LC3-1 electrophoretic mobility and which is referred to as LC3-II. Treatment of LNCaP cells with VMY for 24 hours resulted in an induction of conversion of LC3-I to LC3-II (Fig. 5C). Degradation of the autophagic receptor sequestosome1/p62 [37], together with its autophagic cargo, occurs via the lysosomes during autophagy. Accordingly, treatment with VMY for 24 hrs resulted in a decrease in p62 levels, (Fig. 5C). Collectively, these data clearly suggest that treatment of LNCaP cells with VMY results in an induction of autophagy.

### VMY induces autophagic cell death in LNCaP cells

**Figure 6:**
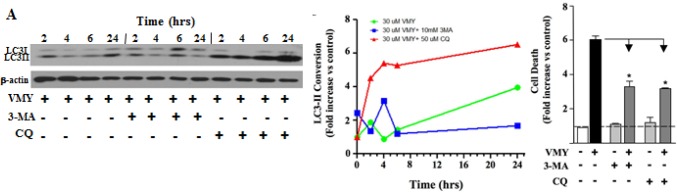
Temporal profile of autophagy induction in LNCaP cells by VMY An extensive assessment of autophagic progression was performed at the times indicated in the presence or absence of the autophagy inhibitors 3-methyladenine (3-MA) and chloroquine (CQ). (A) Induction of autophagy was assessed following treatment with 30 uM VMY. (B) Quantification of normalized protein levels for LC3-II conversion was performed using the protein expression data in DMSO control treated cells (see Supplemental Fig. S2), following normalization to β-actin, allowing the effects of VMY and the independent effects of DMSO, 3-MA or CQ to be directly compared based on the results seen in *A.* The data were plotted as fold change in LC3-II. (C) Effect of inhibition of autophagy on VMY-induced cell death. The fold-increase in cell death induced by VMY in LNCaP cells in the presence or absence of 3-MA or chloroquine was quantified by trypan blue dye exclusion (* *p* < 0.01, N=3).

A static assessment of LC3 conversion fails to clearly indicate whether autophagy is inhibited or activated. To investigate how VMY affects autophagic flux [38], we performed dynamic kinetics experiments where autophagic induction was studied as a function of time following VMY treatment (Fig. 6). In the absence of VMY, the conversion of LC3-I to LC3-II remained relatively stable, with or without 3-MA, and increased slightly after exposure to chloroquine (Supplemental Fig. S3). This result strongly suggests that LNCAP cells have a relatively low rate of basal autophagic flux, at least in the absence of VMY. By contrast, treatment with VMY resulted in a strong induction of LC3-II conversion, as expected (see Fig 5 above), which was blocked by the early autophagy inhibitor 3-MA (Fig. 6A,B (blue line)), and was enhanced by chloroquine (Fig. 6A,B (red line). Importantly, inhibition of autophagy led to an overall reduction in cell death induced by VMY, as measured by trypan blue dye exclusion (Fig. 6C), indicating that the activation of autophagy is a central mechanism in VMY-induced cell death.

### VMY-induced autophagy is p53-dependent

**Figure 7:**
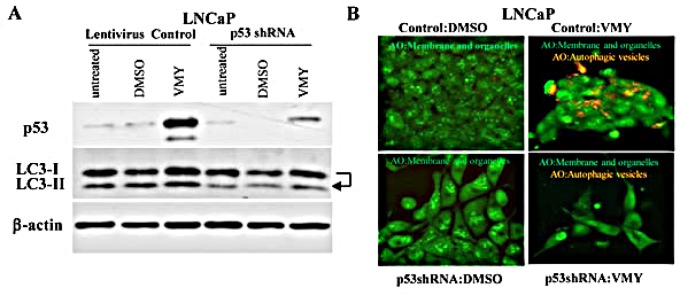
Genetic silencing of p53 inhibits the autophagic response to VMY (A) LNCaP cells were infected with the empty lentivirus control or with the lentivirus p53-shRNA and treated with either DMSO or 30 uM VMY for 18hrs. Western blots were probed for levels of p53 and for LC3-I and LC3-II with β-actin used as a loading control. The arrow highlights the loss of conversion of LC3I to LC3-II, commensurate with p53 knockdown. (B) LNCaP cells infected with the control or p53-shRNA lentivirus's used in panel A and treated for 18 hrs with DMSO (left panels) or 30 uM VMY (right panels) and stained with AO. The AO treated cells were imaged as in Figure 5, and are shown as cytoplasmic- and membrane-associated AO (green, left panels) and as merged images to visualize both cytoplasmic- and membrane-associated AO and protonated AO in acidic vesicles (green and orange, right panels).

In order to establish whether p53 mediates VMY-induced autophagy, we infected LNCaP cells with a previously validated p53 shRNA-expressing lentivirus [39]. p53 shRNA led to an appreciable (greater than 60%) reduction of p53 protein levels compared with vector control (Fig. 7A). VMY failed to activate autophagy in the p53 shRNA-transduced cells, as demonstrated by the lack of conversion of LC3-I to LC3-II (Fig. 7A, arrow). The knockdown of p53 reduced vesicular acidification by VMY (Fig. 7B lower right panel) compared to vector control cells (Fig. 7B upper right panel). Thus, p53 regulates autophagosome formation and functional p53 is required for VMY's induction of autophagy in prostate cancer cells.

### VMY impedes the proliferative capacity of primary prostate cancer cells derived from patients

**Figure 8:**
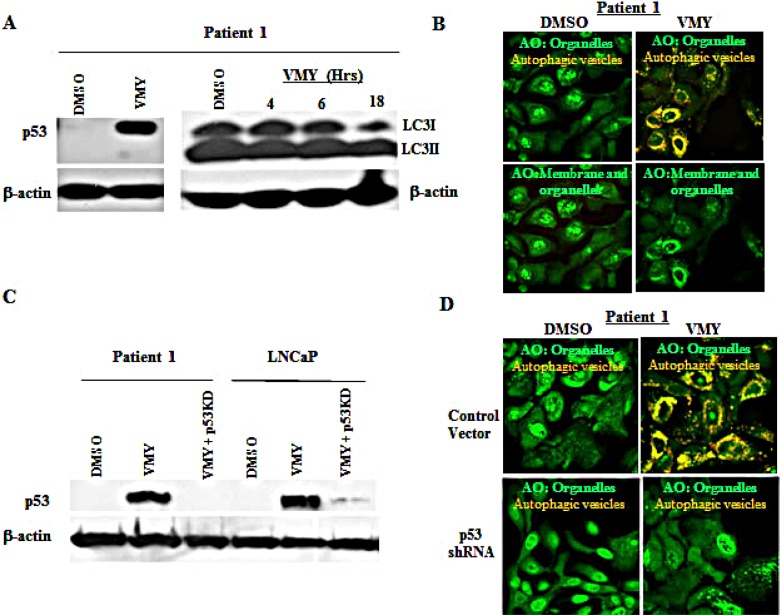
Effects of VMY on prostate cancer conditionally reprogrammed cells (CRCs) (A) The prostate cancer-derived CRCs from Patient 1 were treated with 30 um VMY for 18 hrs and western blotting performed, establishing the induction of p53 protein levels, consistent with results seen in p53 wild-type cell lines (left). The time course of induction of LC3II conversion following treatment with VMY (right). (B) Photomicrographs of cancer CRCs from Patient 1 stained with acridine orange (AO) and treated with DMSO (left panels) or 30 uM VMY (right panels). The AO treated cells were imaged as in Figure 5 and shown merged to visualize cytoplasmic- and membrane-associated AO as well as protonated AO in acidic vesicles (green and orange, top panels) and for only cytoplasmic- and membrane-associated AO (green, bottom panels). (C) Knockdown of p53 (p53shRNA) in the CRCs from Patient 1 by p53-shRNA lentivirus followed by treatment with 30 uM VMY for 18hrs. LNCaP cells were used as a positive control. Western blotting for p53 and β-actin is shown, confirming p53 knockdown. (D) Vector control (top) and p53-shRNA knockdown CRCs (bottom) from Patient 1 were treated with DMSO (left) or 30 uM VMY (right) for 18 hrs, stained with AO and the images merged to visualize cytoplasmic- and membrane-associated AO as well as protonated AO (green and orange).

We have recently reported on a novel cell culture approach that conditionally induces an indefinite proliferative state in primary mammalian epithelial cells [23-25, 40, 41], which are referred to as conditionally reprogrammed cells (CRCs). Prostate cancer CRC lines and their matched normal counterparts were established from two Gleason's grade 7 patients who underwent radical prostatectomy at Massachusetts General Hospital and Georgetown University Hospital. Exposure to VMY for 18hrs established that the tumor CRCs were significantly more sensitive to VMY-induced cell death, compared to the patient-matched normal CRCs, (Supplemental Fig. S4). Cell cycle progression and apoptosis as measured by flow cytometry were also affected (Supplemental Fig. S4). Robust increases in the p53 protein levels were seen following VMY exposure (Fig. 8A), and VMY induced the conversion of LC3I to LC3II by 18hrs versus both early time points and DMSO alone (Fig 8A). In addition, sequencing of the p53 DNA binding domains of both CRC lines revealed no genetic mutations, further confirming that the *p53* genes were indeed wild-type.

VMY also increased the protonated AO fluorescence, with discrete vesicles being observed throughout the cytoplasmic compartment in both CRC lines (Fig. 8B and Supplemental Fig. S5), fully recapitulating our observations in wild-type p53 LNCaP cells. Therefore, to establish whether VMY induction of p53 induces autophagy in prostate cancer CRCs, genetic silencing of p53 by shRNA was next performed. Lentiviral infection of the CRCs was carried out as described above and transduction with the p53shRNA virus resulted in significant reductions in p53 levels in the CRCs (Fig. 8C-left). LNCaP cells were used as a positive control for p53 knockdown (Fig. 8C-right). The genetic silencing of p53 by shRNA significantly reduced VMY-induced vesicular acidification (Fig. 8D, upper right panel vs. lower right panel). These results strongly indicate that the loss of p53 abolishes VMY-induced autophagosome formation in primary prostate cancer cells following treatment.

Taken together, these experiments demonstrate that wild-type p53 is required for VMY-induced autophagy and apoptosis in both transformed cell lines and in primary prostate cancer cells and that VMY-induced cell death is dependent upon p53-driven autophagy.

## DISCUSSION

Apoptosis and autophagy represent two complex and partially overlapping mechanisms that are activated under a variety of cell conditions, including chemotherapeutic stress (see references [38, 42] among others), and where p53 can play a significant regulatory role. For example, the initiation of p53 and apoptosis by chemotherapeutic drugs often results in cell death. Furthermore, while we have recently discovered that autophagy is the key pathways for degrading mutant p53 proteins [20], it is autophagy *per se* that plays a central role in stabilizing wild-type p53 following DNA damage [17]. Additionally, autophagy-related proteins such as Beclin 1, ATG5 [19, 43] and Ambra1 [44] are not only targets of apoptosis but also reciprocally influence apoptotic activity. And, while controlled autophagy can function as a pro-survival mechanism [45], unchecked autophagy can trigger cell death. We predict therefore that compounds that can support p53 induction and both unchecked autophagy and robust apoptosis will deliver increased curative effects.

We had previously reported that VMY induced both p53 protein levels and phosphorylation on serine residues 15, 46 and 393, as well as increasing both p21^CIP1/WAF1^ levels and PARP cleavage [26], suggesting that p53 activity may transduce at least a component of VMY's effects, however this possibility was not directly addressed. Using genetic and chemical approaches, we herein established that wild-type p53 plays an essential role in inducing both apoptosis and autophagy in prostate cancer cells. While there is known crosstalk between autophagy and apoptosis, as they share certain signaling pathways and proteins, the mechanisms by which this bridging occurs have not been fully defined. Furthermore, p53's role in concurrently regulating apoptosis and autophagy differs by cell type and can be influenced by the type of stressor to which the cell is exposed. For example, treatment of SiHa (cervical cancer), A549 (lung) and MCF7 (breast) cancer cells with docosahexaenoic acid resulted in autophagy via an induction of AMPK and mTOR inhibition [46]. However while inhibition of autophagy partially prevented the apoptotic response, the *loss* of p53 was required [46]. In contrast, we show that the induction of p53 by VMY was a prerequisite for inducing both autophagy and apoptosis, and that silencing p53 effectively blocked activation of both pathways. While further studies will be required to more fully elucidate the molecular mechanisms associated with p53's sensitivity to VMY, these studies underscore the complexity of both p53's role in regulating cellular responses to treatment and highlight the importance that the model system plays in both drug development and predictive modeling of therapeutic responses.

The lack of clinically relevant cell lines has had a major impact on effective therapeutic development [47, 48]. By combining detailed tissue collection and pathology with our CRC technology [22-25, 40, 41], we generated two wild-type p53 prostate CRC lines from Gleason score 7 patients. Since the mechanisms by which p53 bridges autophagy and apoptosis to induce cell death are still not fully elucidated, these CRC cultures represent important resources for the assessment of p53 activity in non-transformed prostate cells. For example, we anticipate that prostate cultures derived from local or distant metastases will contain cells harboring mutant *p53* genes [30]. In addition, the establishment of isogenic cell lines derived through the use of gene-targeting vectors and/or viral delivery of shRNAs will allow for the modification of single or multiple genes in either normal or tumor CRCs with minimal impacts on their existing genetic background. Since cultures of CRCs can be established via single cell cloning from the primary tissue sample (CA, XL, RS unpublished), the necessary tools now exist for the in-depth study of p53 function in a highly biologically relevant and patient-specific model system. In addition, the CRC approach allows for the study of inter- and intra- tumor heterogeneity with unprecedented accuracy and detail and will hopefully form the working basis for a more personalized approach to prostate cancer therapy, analogous to the patient we successfully treated for malignant recurrent respiratory papillomatosis, a life threatening cancer of the airway epithelium [24].

In conclusion, we have identified a small molecule-inducible, p53-dependent link to the induction of apoptosis, autophagy and cell death in prostate cancer cells. Interestingly, the co-expression of the p53G245A mutant protein in LNCaP cells failed to interfere with VMY-induced cell death (CA, MA, LR data not shown), suggesting that tumors that are heterozygous for mutant p53 still retain VMY sensitivity. While VMY has not been tested to date in humans, our mouse imaging studies established that VMY (20 mg/kg given three times per week for more than four weeks,) was both an effective treatment for sporadic medulloblastoma and importantly was well tolerated, with no signs of toxicity being found [29]. Furthermore the straightforward mass spectrometry-based method we developed in that study for quantifying systemic VMY delivery to tissues, including the prostate [29], will be useful in facilitating future *in vivo* efficacy and molecular mechanism studies using both engineered mouse models and primary human tissue xenografts. Since PRIMA-1 resensitized p53-mutant cells to VMY-induced cell death, we conclude that this compound may be highly effective in treating a broad array of cancers, including those with p53 mutations. Finally, similar to our recent studies [24, 41], the ability to now perform direct testing of the efficacy of VMY as a monotherapy or in combination with other drugs on individual patients cells may result in more rapidly achievable and more accurate predictions of a patient's response to therapeutic intervention, resulting in better outcomes and enhanced survivorship.

## METHODS

### Cell lines and cell culture

All commercial cell lines were obtained from the ATCC. The human cancer cell lines LNCaP, PC3, AsPC1, PC12 and COLO-357 were maintained in RPMI containing 10% FBS, 1mM sodium pyruvate, and 100 U/ml Penicillin-Streptomycin. The cell lines DU145, MCF7, MDA-MB231, MIA-PACA and A172 were maintained in DMEM media containing 10% FBS, L-glutamine, and 100 U/ml Penicillin-Streptomycin. T98G cells were maintained in Eagle's Minimum Essential Media containing 10% FBS, L-glutamine, and 100 U/ml Penicillin-Streptomycin. Human radical prostatectomy samples were collected under the auspices and approval of the Georgetown University and Massachusetts General Hospital Institutional Review Boards. Following detailed pathological analyses that documented that the tissue sections collected were nearly exclusively tumor cells, the specimens were processed via protease dissociation as previously described [23]. Primary cultures were established at Georgetown using the CRC method as previously described [23]. For experimentation, the cells were carried in conditioned media as described [25]. Briefly, irradiated J2 feeder cells were plated in 175 cm^2^ tissue culture flasks (BD Biosciences, Franklin Lakes, NJ) in 30 mls of F media. The media was collected after three days in culture and centrifuged at 1000 x *g* for 5 minutes at 4^°^C to remove cellular debris, followed by filtration using a 0.22 μ;m Millex-GP filter unit (Millipore, Billerica, MA). For cell culture, three volumes of conditioned F media was mixed with one volume of fresh F medium and the final working conditioned media was supplemented with 5 μ;M Y-27632.

### Flow cytometry

The prostate cells were fixed and stained with 20ug/ml propidium iodide (PI) and 5 U RNase A, and the DNA content and SubG1 DNA fragmentation was measured using a FACStar Plus system (Becton-Dickson, Franklin Lakes, NJ) as previously described [26, 27]. Cellular apoptosis was also assessed by APC-Annexin V antibody (Biolegend, San Diego, CA) staining immediately after treatment with VMY and analyzed using FACStar Plus dual laser FACSort system (Becton-Dickson, Franklin Lakes, NJ) as previously described by us [26, 27, 49, 50].

### Immunoblotting

Protein extracts were separated on 4-20% Tris-glycine gels and electro-blotted onto PVDF membranes as previously described [13, 26, 27]. Protein levels were assessed using antibodies against p53 (Millipore, #05-224), PARP (Cell Signaling, Danvers, MA #9542), b-actin (Cell Signaling, Danvers, MA #4967), p21 (Santa Cruz #SC 756), LC3B (Cell Signaling, Danvers, MA #3868S) and P62 (Cell Signaling, Danvers, MA #5114S) Densitometry was performed using ImageJ analysis software (NIH, Bethesda, MD) as previously described [13, 26, 27].

### Cell viability and growth

Cell viability was determined using trypan blue dye exclusion and viable and total cell counting using a hemocytometer as previously described [26, 27].

### p53 expression and shRNA knockdown

The p53 wild-type and p53G245A mutant vectors, have been previously described by us [14, 51]. For adenovirus knockdown experiments, Adp53shRNA and empty vector control virus were purchased commercially (Vector Biolabs, Philadelphia, PA, #1854) and used as described by the manufacturer. For lentivirus-based p53 knockdown experiments, the pLKO-p53shRNA and empty vector lentivirus vectors (kindly provided by Todd Waldman, Georgetown University) were used as described [39]. Briefly, 293T cells (ATCC, Manassas, VA) were cotransfected with shRNA constructs along with the pHR′8.2ΔR and pCMV-VSV-G helper constructs. After 24 hours, the media was changed and the virus-containing media was harvested after an additional 24 hours of incubation. The prostate cancer cells were seeded at 30% confluency and viral infections were performed for 72 hours prior to treatment with VMY or DMSO. Efficiency of the knockdown was monitored by p53 immunoblotting as previously described [14, 26, 51].

### p53 restoration

DU145 cells were pre-treated with 75μ;M PRIMA-1 (Sigma, St Louis, MO) for 24hrs prior to treatment. Activity of PRIMA-1 was monitored via assessing the induction of p21^CIP1/WAF1^ (Santa Cruz, sc481) protein levels by immunoblot.

### Autophagy and Apoptosis Inhibitors

For the autophagy inhibition, 3-methyladenine (3-MA) (Sigma, St Louis, MO M921) was used at 5mM and 10 mM concentrations and chloroquine diphosphate (Sigma, St Louis, MO) was used at 50 μ;M and 100 μ;M. Cells were exposed to these inhibitors for 20 minutes prior to treatment with either DMSO or VMY.

The caspase 8 inhibitor Z-IETD-FMK, (Sigma, St Louis, MO) and the caspase 9 inhibitor Z-LEHD-FMK (Sigma, St Louis, MO) were resuspended in DMSO and used at final concentrations of 20uM.

### Autophagic vesicle maturation

Autophagic vesicle maturation was analyzed by the detection of acidic vesicular organelles and LC3-GFP localization. For both the experiments, LNCaPs, DU145 and PC3 cell lines were analyzed.

In order to detect the acidic vesicles in presence or absence of VMY treatment, the cells were stained with acridine orange (AO). Briefly, the cells were grown overnight on World Precision Instruments 35 mm glass Fluorodish cell culture dish (Fisher Scientific, Waltham, MA) in appropriate media. After an 18 hr incubation with 30 μ;M VMY, the AO solution was added at a final concentration of 10 μ;M, and the cells were incubated for 20 minutes at 37ºC. Live cells were imaged on a Zeiss (Thornwood, NY) LSM510 Meta microscope_._ Non-protonated AO (green fluorescence) was imaged using argon 488 laser and a 488 dichroic mirror and a 525/50 bandpass filter. For the protonated (orange/red fluorescence) species of AO, a multi-photon chameleon laser was set to 760 with a 700 short pass filter using a 651/42 emission filter. LC3 translocation was detected using the green fluorescent protein (GFP)-fused LC3 construct that was generously donated by Dr Robert Clarke [36]. Briefly, cells were seeded in 6 well plates contained glass coverslips and allowed to attach overnight. 14 ug of LC3-GFP expression plasmid were transfected using Lipofectamine LTX reagent (Life Technologies, Carlsbad, CA) as previously described [36]. 24 hours after transfection, the cells were pretreated or not with 5uM 3MA or 50 uM Chloroquine for 20 minutes and further treated with VMY or vehicle. After 18 hours, the coverslips with attached cells were stained with DAPI and rinsed 3 times with PBS. The excess buffer was removed and the coverslips were mounted. Imaging was performed by confocal microscopy as previously described [27].

## SUPPLEMENTARY MATERIAL FIGURES


