# Supplementary material for: The induction of the p53 tumor suppressor protein bridges the apoptotic and autophagic signaling pathways to regulate cell death in prostate cancer cells
Source: Oncotarget. 2014 Sep 26;5(21):10678–91. doi: 10.18632/oncotarget.2528 (PMC4279402; doi:10.18632/oncotarget.2528)
Supplement: Supplementary file 1 [file oncotarget-05-10678-s001.pdf]

The induction of the p53 tumor suppressor protein bridges the apoptotic and autophagic signaling pathways to regulate cell death in prostate cancer cells

Supplementary Material

A

LNCaP (p53 Wt)

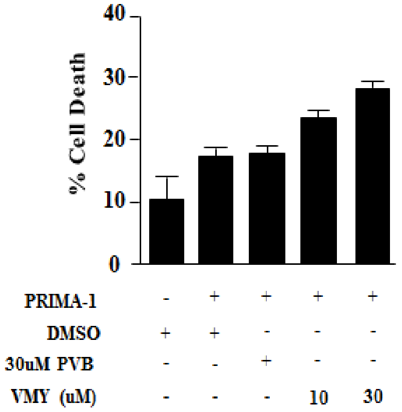

B

PC3 (p53 null)

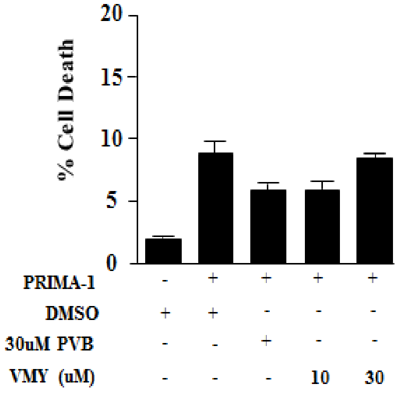

Fig S1: PRIMA-1 does not influence VMY sensitivity in **A)** p53 WT and **B)** p53 null prostate cancer cells. Both cell types were treated with 75 uM PRIMA-1 and either VMY at 10uM or 30uM or 30 uM PVB (purvalanol B) at 30uM for 18 hrs. Cell viability was measured by trypan blue dye exclusion.

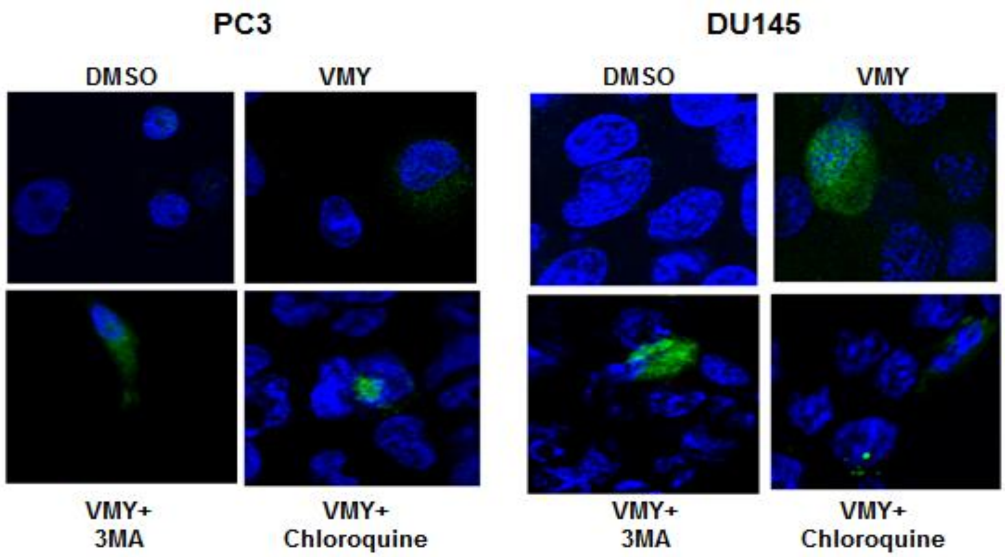

Fig S2: VMY fails to induce autophagy in PC3 (Left) and DU145 cells (Right) as measured by LC3-GFP fluorescence. 3MA, 3-methyladanine

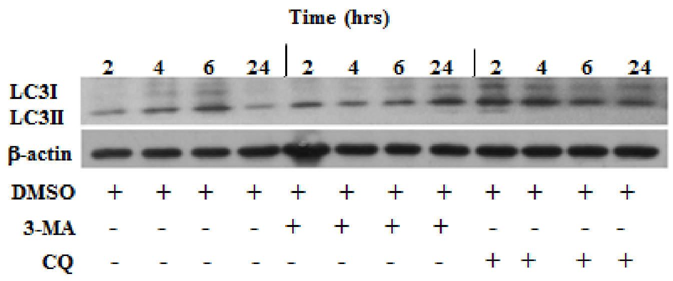

Fig S3: Basal levels of authophagic proteins in DMSO control cells. The effects of 5 uM 3-methyladenine(3-MA) or 50 uM chloroquine (CQ) on the autophagic markers p62 and LC3I/II, were assessed in LNCaP cells. Samples were collected at the times shown. The membranes were probed for p62 and LC3I/II, with β-actin as a loading control.

A

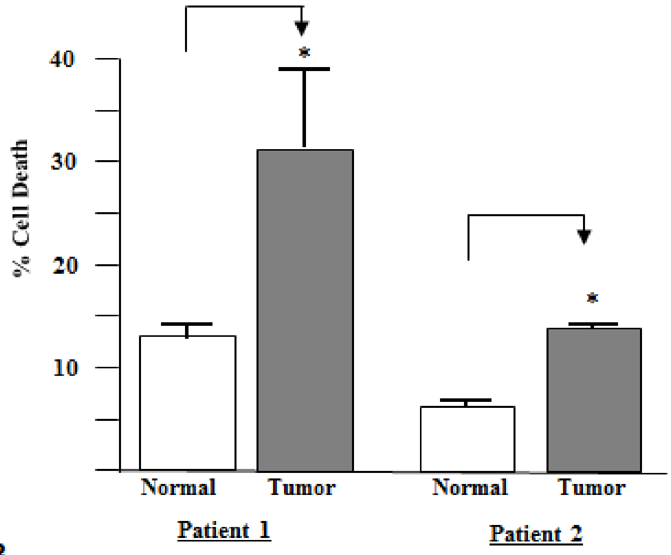

B

Patient 1: Tumor Derived CRCs

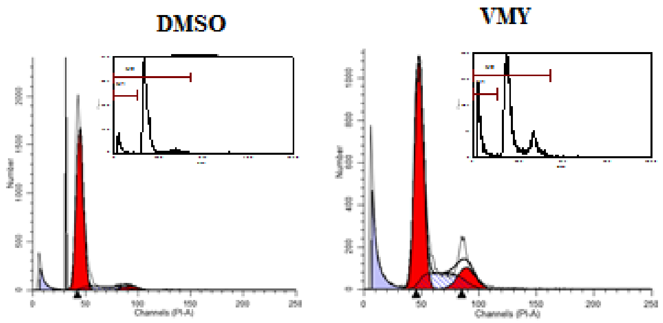

(%)	Treatment	
	DMSO	VMY (30uM)
G1	86	67
S	10	20
G2/M	4	13
subG1	9	20
annexin V	1.6	5.7

Patient 2: Tumor Derived CRCs

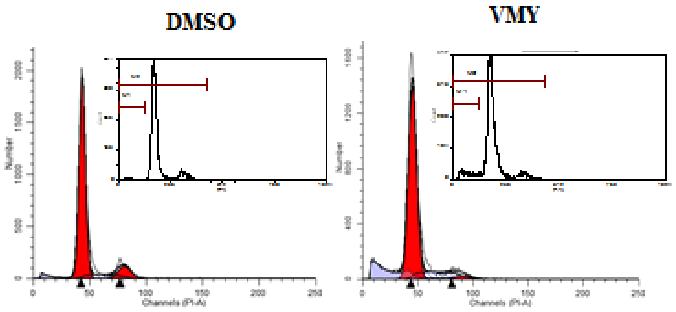

(%)	Treatment	
	DMSO	VMY (30uM)
G1	82	82
S	8	15
G2/M	10	3
subG1	3	10
annexin V	2.0	4.4

Fig S4: Effects of VMY on CRCs from Patients 1 and 2. **A)** Cell viability as measured by trypan blue of patient-matched CRCs following 18hr exposure to 30uM VMY. **B)** Cell cycle effect of 18hr exposure to 30uM VMY as measured by flow cytometry. * $p < 0.05$.

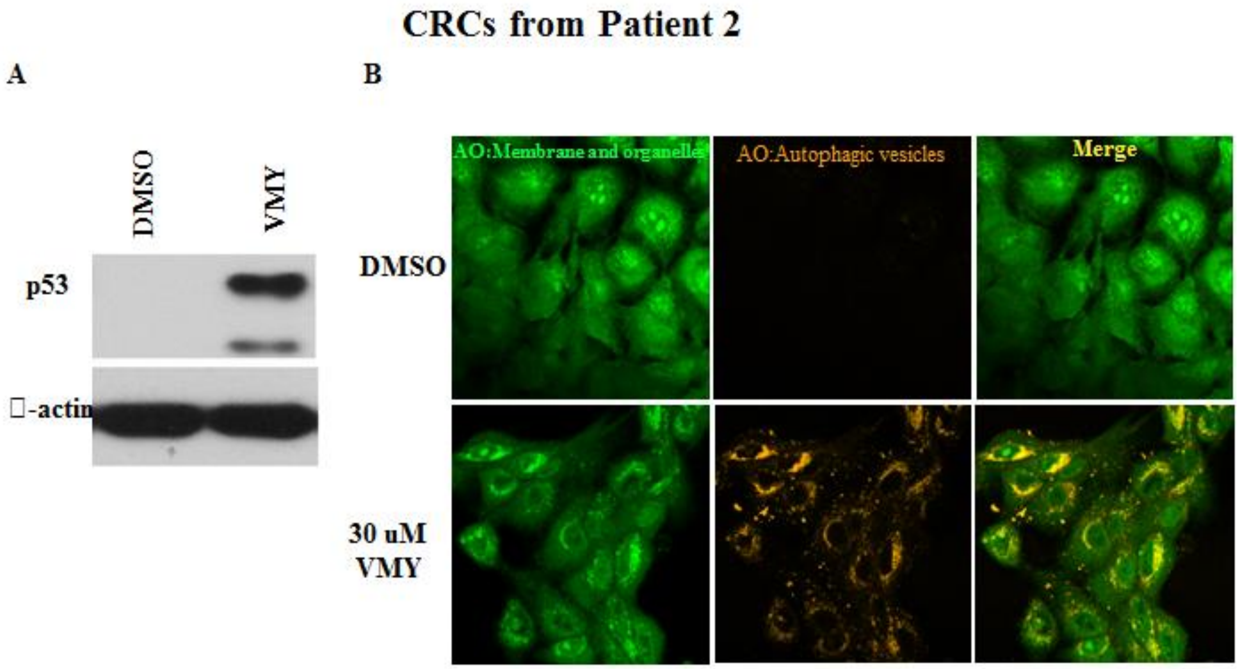

Fig S5: Induction of p53 and autophagy in primary prostate cancer CRCs from Patient 2 tretaed with 30uM VMY for 18 hours. **A)** Westen blot showing induction of p53 levels by VMY. **B)** Photomicrographs of LNCaP cells stained with acridine orange (AO) and treated with DMSO (top row) or 30 uM VMY (bottom row) for 18 hrs.The cells were imaged on a Zeiss LSM510 Meta microscope using a 488 nm dichroic mirror and a 525/50 bandpass filter to visualize cytoplasmic- and membrane- associated AO (green, left panels) and a 700 nm short pass filter using a metadetector of 651/42 emission filter to visualize protonated AO in acidic vesicles (orange, middle panels) and as merged images (green and orange, right panels)
